# Preditores de *clusters* de trajetórias de intensidade de atividade física no lazer em homens e mulheres do ELSA-Brasil

**DOI:** 10.1590/0102-311XPT132924

**Published:** 2025-05-19

**Authors:** André Luis Messias dos Santos Duque, Daniela Polessa Paula, Francisco José Gondim Pitanga, Ciro Oliveira Queiroz, Maria del Carmen Bisi Molina, Alexandra Dias Moreira, Maria da Conceição Chagas de Almeida, Sheila Maria Alvim de Matos, Ana Luísa Patrão, Maria de Jesus Mendes da Fonseca, Rosane Harter Griep

**Affiliations:** 1 Instituto Nacional de Cardiologia, Rio de Janeiro, Brasil.; 2 Prefeitura Municipal de Petrópolis, Petrópolis, Brasil.; 3 Escola Nacional de Ciências Estatísticas, Rio de Janeiro, Brasil.; 4 Universidade do Estado do Rio de Janeiro, Rio de Janeiro, Brasil.; 5 Universidade Federal da Bahia, Salvador, Brasil.; 6 Escola Bahiana de Medicina e Saúde Pública, Salvador, Brasil.; 7 Universidade Federal do Espírito Santo, Vitória, Brasil.; 8 Universidade Federal de Minas Gerais, Belo Horizonte, Brasil.; 9 Instituto Gonçalo Moniz, Fundação Oswaldo Cruz, Salvador, Brasil.; 10 Centro de Psicologia, Universidade do Porto, Porto, Portugal.; 11 Fundação Oswaldo Cruz, Rio de Janeiro, Brasil.

**Keywords:** Atividade Física, Trajetória de Vida, Fatores Sociodemográficos, Estilo de Vida, Physical Activity, Life Experience, Sociodemographic Factors, Life Style, Actividad Física, Experiencias de Vida, Factores Sociodemográficos, Estilo de Vida

## Abstract

A manutenção da atividade física ao longo do tempo é um desafio para a saúde pública. Preditores de diferentes intensidades de atividade física não foram suficientemente analisados. Este estudo objetivou identificar *clusters* de trajetórias de intensidade de atividade física no lazer, seus preditores e o perfil dos participantes situados nos *clusters*. Incluíram-se dados da linha de base e duas visitas de acompanhamento de 11.262 participantes do Estudo Longitudinal de Saúde do Adulto (ELSA-Brasil). A atividade física foi avaliada em três momentos do tempo por meio do *Questionário Internacional de Atividade Física* (IPAQ, acrônimo em inglês). Identificaram-se *clusters* de trajetórias de atividade física segundo intensidade (fraca, moderada e forte) a partir do K-means longitudinal. A escolha do número de *clusters* baseou-se na medida *within-clusters sum-of-squares* (WCSS) e a classificação tiveram como base as recomendações científicas. Utilizou-se o *machine learning* para verificar a importância dos preditores. Identificaram-se cinco *clusters* para os homens e quatro para as mulheres. Os homens do *cluster* adequado com aumento de atividade física forte tinham maior renda, escolaridade e consumo diário de frutas e verduras; eram mais jovens; nunca haviam fumado e tinham um estado nutricional normal. Já as mulheres do *cluster* adequado com aumento de atividade física moderada tinham maior renda e escolaridade; nunca haviam fumado e o estado nutricional delas era normal. Em ambos os sexos, idade e escolaridade foram os preditores de maior importância para a classificação nos *clusters*. Devem ser implementadas ações promotoras de atividade física ao longo do tempo, adaptadas a fatores sociodemográficos e comportamentais.

## Abstract (en)

The maintenance of physical activity over time is a challenge for public health. Predictors of different physical activity intensities have not been sufficiently analyzed. This study aimed to identify clusters of trajectories of physical activity intensity in leisure time, their predictors and the profile of the participants in the clusters. Baseline data and two follow-up visits of 11,262 participants from the *Brazilian Longitudinal Study of Adult Health* (ELSA-Brazil) were included. physical activity was assessed at three moments using the *International Physical Activity Questionnaire* (IPAQ). Clusters of physical activity trajectories according to intensity (weak, moderate and strong) were identified via longitudinal K-means. The number of clusters was based on the within-clusters sum-of-squares (WCSS) measure and the classification was based on scientific recommendations. Machine learning was used to verify predictors importance. Five clusters were identified for men and four for women. Men in the adequate cluster with a strong increase in physical activity had higher income, schooling level, and daily consumption of fruits and vegetables; they were younger; had never smoked and had a normal nutritional status. On the other hand, women in the adequate cluster with moderate physical activity increase had higher income and schooling level; had never smoked and had a normal nutritional status. In both sexes, age and schooling level were the most important predictors for classification in clusters. Actions to promote physical activity should be implemented over time, and be adapted to sociodemographic and behavioral factors.

## Abstract (es)

El mantenimiento de actividad física a lo largo del tiempo es un desafío para la salud pública. Los predictores de diferentes intensidades de actividad física no están evaluados de manera suficiente. Este estudio tuvo como objetivo identificar los clústeres de trayectorias de intensidad de actividad física en el ocio, sus predictores y el perfil de los participantes situados en los clústeres. Se incluyeron datos de referencia y dos visitas de seguimiento a 11.262 participantes del *Estudio Longitudinal de Salud de Adultos* (ELSA-Brasil). La actividad física se evaluó en tres momentos mediante el uso del *Cuestionario Internacional de Actividad Física* (IPAQ, por su sigla en inglés). Se identificaron clústeres de trayectorias de actividad física según intensidad (débil, moderada y fuerte) mediante el K-means longitudinal. La elección del número de clústeres se basó en la medida *within-clusters sum-of-squares* (WCSS), y la clasificación se basó en recomendaciones científicas. Se utilizó el aprendizaje automático para verificar la importancia de los predictores. Se identificaron cinco clústeres para hombres y cuatro para mujeres. Los hombres en el clúster adecuado con un sólido aumento de actividad física tenían un mayor ingreso, nivel de estudios y consumo diario de frutas y verduras; eran más jóvenes; nunca habían fumado y tenían un estado nutricional normal. Por otro lado, las mujeres en el clúster adecuado con un aumento en la actividad física moderada tenían mayor ingreso y nivel de estudios; nunca habían fumado y tenían un estado nutricional normal. En ambos sexos, la edad y el nivel de estudios fueron los predictores más importantes para la clasificación en clústeres. Las acciones que promuevan la actividad física deben implementarse a lo largo del tiempo, con adaptación a los factores sociodemográficos y de comportamiento.

## Introdução

A manutenção da atividade física está associada a muitos benefícios à saúde, entre os quais se encontra um menor risco de doenças cardiovasculares, câncer, diabetes, obesidade, depressão e mortalidade por todas as causas ^1^ ^,^ ^2^, sendo fundamental para a longevidade ^3^ ^,^ ^4^ ^,^ ^5^. A atividade física pode ser praticada em diferentes intensidades, e a recomendação é que sejam realizados entre 150 a 300 minutos por semana em intensidade moderada, 75 a 150 em intensidade forte ou, ainda, uma combinação de ambas ^6^. A intensidade de atividade física representa a sobrecarga imposta ao organismo, sendo essencial para a manutenção da saúde ^7^.

Todavia, a manutenção da intensidade recomendada ao longo do tempo apresenta níveis inadequados ^8^. No Brasil, de acordo com estudo populacional feito com 88.531 indivíduos da *Pesquisa Nacional de Saúde* de 2019, somente 19,4% dos brasileiros atenderam às recomendações para atividade física no tempo livre, sendo as mulheres (63%) mais inativas do que os homens (55,5%) ^9^. Isso constitui um desafio para a saúde pública, tornando necessário investigar longitudinalmente preditores da prática e manutenção da atividade física ^10^.

Um estudo observacional realizado na Inglaterra, incluindo dados de 7.735 homens recrutados entre 1978 e 1980 e que foram acompanhados após 12, 16 e 20 anos, demonstrou que idade, situação conjugal, tabagismo, consumo de álcool e hábitos alimentares são preditores de trajetórias da atividade física, e deveriam ser considerados ao se investigar a prática da atividade física ao longo do tempo ^11^. Em revisão sistemática, Lounassalo et al. ^10^ utilizaram dados de artigos publicados entre 2000 e 2018 que identificaram trajetórias longitudinais de atividade física e seus preditores e concluíram que sexo, renda e escolaridade são importantes fatores associados à manutenção da atividade física ao longo do tempo. Além disso, os resultados disponíveis na literatura sobre trajetórias de atividade física entre homens e mulheres são inconclusivos a respeito dos níveis de atividade física ao longo do tempo ^10^ ^,^ ^11^ ^,^ ^12^ ^,^ ^13^. Sendo assim, são necessários novos estudos longitudinais que analisem de forma estratificada por sexo a prática de atividade física ao longo do tempo.

Ressalta-se, ainda, que a maioria dos estudos que avaliaram os fatores sociodemográficos e comportamentais associados a trajetórias da atividade física foram realizados em países de alta renda, com populações jovens, tempos curtos de avaliação e não consideraram as diferentes intensidades da atividade física ^4^ ^,^ ^10^ ^,^ ^11^ ^,^ ^14^ ^,^ ^15^ ^,^ ^16^ ^,^ ^17^ ^,^ ^18^, gerando lacunas de conhecimento. Os achados de novos estudos podem contribuir com a implementação de políticas públicas que promovam a atividade física ao longo do tempo dentro da intensidade recomendada. Portanto, o objetivo deste estudo foi identificar *clusters* de trajetórias de intensidade de atividade física no lazer, seus preditores e o perfil dos participantes situados nos *clusters*.

## Método

### Desenho de estudo

Estudo longitudinal utilizando dados do *Estudo Longitudinal da Saúde do Adulto* (ELSA-Brasil), uma coorte prospectiva e multicêntrica desenvolvida em seis instituições de ensino superior e pesquisa das regiões Nordeste, Sul e Sudeste do Brasil, com foco na incidência de doenças cardiovasculares, diabetes e fatores de risco associados. Na linha de base, o ELSA-Brasil inscreveu 15.105 servidores públicos ativos e aposentados, com idades entre 35 e 74 anos.

A coleta de dados inicial ocorreu entre 2008 e 2010. Entre 2012 e 2014, ocorreu a segunda coleta de dados, e entre 2016 e 2018, foi feita a terceira coleta de dados. Foram realizados exames clínicos, antropométricos e entrevistas detalhadas por meio de questionários. As coletas de dados ocorreram nos Centros de Investigação ELSA-Brasil de cada instituição, sendo realizadas por meio de técnicas padronizadas feitas por pesquisadores treinados e certificados ^19^ ^,^ ^20^ ^,^ ^21^.

### Participantes

Foram considerados elegíveis para esta análise os participantes que responderam aos questionários sobre atividade física nos três momentos do tempo. Foram excluídos óbitos ocorridos ao longo do tempo, participantes com dados não plausíveis sobre a prática da atividade física (> 840 minutos/semana da atividade física fraca, > 630 minutos/semana da atividade física moderada e > 420 minutos/semana da atividade física forte) ^22^ e participantes que apresentavam dados incompletos para as variáveis sociodemográficas e comportamentais analisadas.

O ELSA-Brasil foi aprovado pela Comissão Nacional de Ética em Pesquisa (Conep) e pelos Comitês de Ética em Pesquisa dos seis centros de investigações envolvidos (Instituto de Saúde Coletiva, Universidade Federal da Bahia - 0017.1.069.000-06; Fundação Oswaldo Cruz - 0058.0.011.000-07; Hospital Universitário, Universidade de São Paulo - 0016.1.198.000-06; Universidade Federal de Minas Gerais - 0186.1.203.000-06; Centro de Ciências da Saúde, Universidade Federal do Espírito Santo - 08109612.7.2003.5060; e Hospital de Clínicas de Porto Alegre, Universidade Federal do Rio Grande do Sul - 48608515.5.1001.5327). Todos os participantes assinaram o termo de consentimento livre e esclarecido, sendo garantidos o sigilo e a confidencialidade dos dados. Este estudo foi aprovado pelos centros de pesquisa envolvidos do ELSA-Brasil, e todos os participantes assinaram o termo de consentimento livre e esclarecido.

### Variáveis

A variável desfecho do estudo é a trajetória de intensidade de atividade física. Para avaliar a atividade física, foi utilizado o domínio da atividade física no tempo livre do *Questionário Internacional de Atividade Física* (IPAQ, acrônimo em inglês), versão longa ^23^. Esse domínio é composto por questões relacionadas à frequência, intensidade e duração da atividade física, mensurada em minutos/semana por meio da multiplicação da duração de cada uma das atividades realizadas pela frequência semanal, e foram contempladas atividades de intensidade fraca, moderada e forte. A atividade física de intensidade fraca se refere a atividades que exigem pouco esforço do organismo (praticamente não gerando alterações na frequência respiratória quando comparada ao repouso), a atividade física de intensidade moderada gera um aumento moderado na frequência respiratória e atividade física de intensidade forte exige grande esforço do organismo ^23^. A aferição de atividade física nos três momentos de coleta permitiu descrever a trajetória de atividade física de cada participante, considerando sua intensidade e modificação no tempo. Para a classificação de atividade física, foi utilizada a recomendação da Organização Mundial da Saúde (OMS), da prática de 150 a 300 minutos por semana de atividade física em intensidade moderada, 75 a 150 minutos em intensidade forte ou ainda uma combinação de ambas ^6^. Portanto, para a análise da trajetória de atividade física de cada indivíduo, foram avaliados o tempo semanal em cada um dos três momentos de coleta de dados e as alterações (aumento, diminuição, manutenção) ocorridas ao longo do tempo. A intensidade fraca foi utilizada para a identificação dos *clusters*, mas não foi considerada para a classificação das trajetórias de atividade física.

### Variáveis sociodemográficas e comportamentais

As variáveis de exposição utilizadas foram: sexo (feminino e masculino), faixa etária (35-44, 45-54, 55-64 e ≥ 65 anos), renda *per capita* (contínua), ocupação (ativo e aposentado), escolaridade (alta escolaridade: nível Superior e baixa escolaridade: até nível Médio completo), situação conjugal (casado, incluindo união estável e não casado, incluindo divorciado, solteiro e viúvo) e tabagismo (ex-fumante, fumante e nunca fumou). O consumo de álcool foi obtido a partir de duas perguntas que avaliavam o consumo de bebidas alcoólicas, sendo os participantes classificados em consumo atual, no passado ou nunca consumiram. O consumo diário de frutas e de verduras foi obtido a partir de duas perguntas que avaliavam a frequência com que os participantes consumiam verduras e legumes crus ou frutas, com categorias de resposta variando de nunca/quase nunca a mais de três vezes por dia, sendo classificados “sim’’ aqueles que referiram consumir uma vez por dia ou mais e “não’’ aqueles que não consumiam diariamente. O estado nutricional foi obtido por meio do índice de massa corporal (IMC), e os participantes foram classificados como: peso normal (IMC ≤ 24,9kg/m^2^, sobrepeso (IMC entre 25 e 29,9kg/m^2^) e obesos (IMC ≥ 30kg/m^2^). Todas as informações foram coletadas na linha de base do ELSA-Brasil por meio de avaliação antropométrica e questionário estruturado em entrevista presencial.

### Análise de dados

As análises foram estratificadas por sexo e realizadas no software R versão 4.2.2 (http://www.r-project.org) e Phyton versão 3.10 (http://www.python.org). Foi realizada análise descritiva da amostra por meio da distribuição de frequências absolutas e percentuais para variáveis categóricas e de média e desvio padrão (DP) para a variável contínua. Para identificar os *clusters* das trajetórias de intensidade da atividade física, foi utilizado o K-means longitudinal, por meio do pacote *kml3D* (https://cran.rproject.org/web/packages/kml3d/index.html), do software R, e o número de *clusters* foi determinado considerando o *within-clusters sum-of-squares* (WCSS), que é a soma de quadrados dentro do *cluster*, podendo também ser chamada de entropia, que mede a distância média quadrada de todos os pontos dentro de um *cluster* até o centroide dele. O número de *clusters* foi determinando considerando a menor distância WCSS. Para comparar as proporções dos participantes em cada *cluster*, foram realizados o teste qui-quadrado (variáveis categóricas) e o teste t de Student para a variável contínua, considerando um nível de 5% de significância.

Para a identificação dos preditores dos *clusters* das trajetórias da intensidade da atividade física, foi implementada a floresta aleatória multiclasse, que utiliza um conjunto de decisões para realizar tarefas de classificação ^24^.

Em função das diferenças existentes entre os *clusters*, as medidas de desempenho ponderadas (acurácia, precisão, revocação e F1-*score*) foram calculadas por meio de validação cruzada para obter a confiabilidade da interpretação ^24^. Os resultados encontrados estão em Material Suplementar (Tabela S1, https://cadernos.ensp.fiocruz.br/static//arquivo/suppl-e00132924_9759.pdf).

Para verificar a influência de cada preditor para a classificação em cada *cluster* e obter a interpretação dos resultados, utilizou-se a abordagem *SHapley Additive exPlanations* (SHAP), uma ferramenta poderosa para extrair a importância das variáveis para a classificação, bem como a direcionalidade de seu efeito ^25^. Para visualizar a influência dos preditores nos *clusters*, foram utilizados os valores SHAP de importância global e local, para determinar a importância global dos recursos para a previsão e para avaliar como essa influência muda dependendo da magnitude dos valores (mais baixos ou mais altos) observados nos recursos (importância local) ^26^. Na análise gráfica de importância local, o eixo y indica a importância do preditor para a classificação, ordenada do maior para o menor, e o eixo x, o valor do SHAP. Para cada variável no eixo y, os pontos espalhados na direção do eixo x representam as observações individuais dos participantes, indicando que para um determinado preditor, a coordenada de um ponto no eixo x é o valor SHAP para o participante. Ou seja, a influência do valor do preditor para a direcionalidade da classificação do participante no *cluster* ^26^.

## Resultados

**Figura 1:**
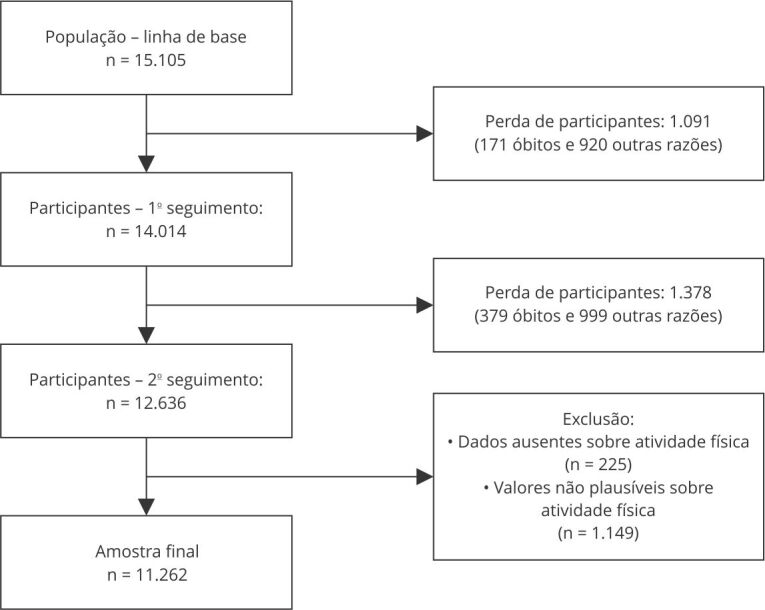
Critérios de seleção da amostra do estudo. *Estudo Longitudinal de Saúde do Adulto* (ELSA-Brasil), 2008-2019.

De um total de 15.105 participantes da linha de base do ELSA-Brasil, 12.636 atenderam ao critério de inclusão (apresentar dados sobre atividade física nos três momentos). Após a exclusão de participantes com dados faltantes e valores não plausíveis sobre atividade física, a amostra final foi composta por 11.262 indivíduos, sendo 6.370 (56,6%) mulheres e 4.892 (43,4%) homens (Figura 1).

Comparadas aos homens, as mulheres referiram renda mais alta, eram mais frequentemente aposentadas, não casadas, nunca fumaram ou consumiram álcool e referiram maior frequência do consumo diário de verduras e frutas. Embora entre elas a prevalência de obesidade tenha sido mais alta, os homens tinham prevalência mais alta de sobrepeso (Tabela 1).

**Tabela 1:** Distribuição proporcional das variáveis sociodemográficas e comportamentais segundo sexo. *Estudo Longitudinal de Saúde do Adulto* (ELSA-Brasil), 2008-2019.

Variáveis	Mulheres (%) [N = 6.370]	Homens (%) [N = 4.892]
População	56,6	43,4
Renda *per capita* (média ± DP)	1.944,85 ± 1.485,1	1.762,54 ± 1.333,2
Faixa etária (anos)		
35-44	22,8	24,8
45-54	41,4	40,4
55-64	27,9	25,6
≥ 65	7,9	9,2
Ocupação		
Aposentado	20,7	13,9
Ativo	79,3	86,1
Escolaridade		
Baixa escolaridade	44,1	46,2
Alta escolaridade	55,9	53,8
Situação conjugal		
Casado	54,4	82,2
Não casado	45,6	17,8
Tabagismo		
Ex-fumante	25,2	34,8
Fumante	11,5	12,5
Nunca fumou	63,3	52,7
Consumo de álcool		
No passado	19,5	19,2
Atual	65,8	74,7
Nunca consumiu	14,7	6,1
Estado nutricional		
Normal	39,5	34,4
Sobrepeso	35,9	45,1
Obeso	24,6	20,5
Consumo diário de verduras		
Sim	57,3	46,1
Não	42,7	53,9
Consumo diário de frutas		
Sim	64,5	47,6
Não	35,5	52,4

DP: desvio padrão.

**Figura 2:**
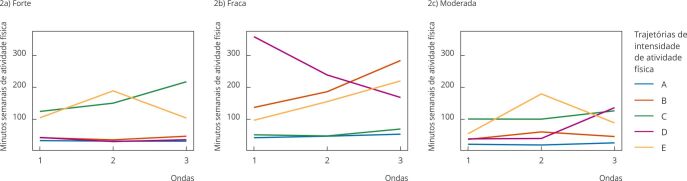
*Clusters* de trajetórias de intensidade de atividade física para homens. *Estudo Longitudinal de Saúde do Adulto* (ELSA-Brasil), 2008-2019.

As Figuras 2 e 3 apresentam os *clusters* para homens e mulheres, respectivamente. Em relação aos homens, foram identificados cinco *clusters* das trajetórias de intensidade de atividade física. O *cluster* A agrupou participantes que, ao longo das três ondas, se mantiveram em poucos minutos de atividade física forte, fraca ou moderada, e foi denominado “insuficiente constante”. O B agrupou aqueles que aumentaram o tempo de atividade física fraca, mas mantiveram pouco tempo de atividade física moderada ou forte, denominado “insuficiente, com aumento da atividade física fraca”. O C, embora tenha poucos minutos de atividade física fraca, incluiu participantes com tempo recomendado para atividade física moderada, sendo estável no tempo e aumentando a atividade física forte, sendo denominado “adequado, com aumento de atividade física forte”. O D incluiu o grupo que reduziu a atividade física fraca, aumentou o tempo de atividade física moderada, mas de forma insuficiente, e manteve poucos minutos dedicados à atividade física forte, denominado “insuficiente, com redução de atividade física fraca e aumento da moderada”. Por fim, o *cluster* E incluiu participantes que aumentaram o tempo dedicado à atividade física fraca, oscilaram no tempo dedicado às atividade física moderada e forte, com tendência a redução das intensidades moderadas e forte, foi denominado “adequado, oscilando atividade física moderada e forte”. Observou-se que os homens situados no *cluster* C apresentaram o dobro de tempo de atividade física em intensidade forte quando comparados aos participantes do *cluster* E (Figura 2).

Nota: insuficiente constante (*cluster* A); insuficiente, com aumento da atividade física fraca (*cluster* B); adequado, com aumento da atividade física forte (*cluster* C); insuficiente, com redução de atividade física fraca e aumento da moderada (*cluster* D); adequado, oscilando atividade física moderada e forte (*cluster* E).

**Figura 3:**
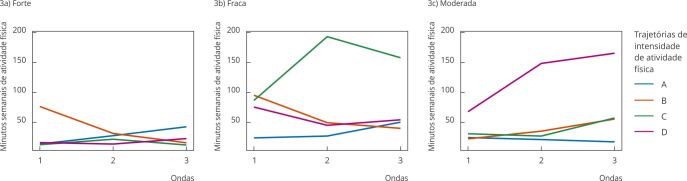
*Clusters* de trajetórias de intensidade de atividade física para mulheres. *Estudo Longitudinal de Saúde do Adulto* (ELSA-Brasil), 2008-2019.

Para as mulheres (Figura 3), identificaram-se quatro *clusters* de trajetórias de intensidade da atividade física. O A agrupou participantes que, ao longo das três ondas, se mantiveram em poucos minutos de atividade física fraca, moderada ou forte, e foi denominado “insuficiente constante”. O B agrupou mulheres que aumentaram o tempo de atividade física moderada, mas mantiveram pouco tempo de atividade física moderada ou forte, denominado “insuficiente, com aumento de atividade física moderada”. O C incluiu grupo com tempo insuficiente para atividade física moderada e forte e oscilação de atividade física fraca e foi denominado “insuficiente, oscilando atividade física fraca”. E o D incluiu participantes que aumentaram o tempo na atividade física moderada e mantiveram poucos minutos na atividade física fraca e forte e foi denominado “adequado, com aumento de atividade física moderada”.

Nota: insuficiente constante (*cluster* A); insuficiente, com aumento da atividade física moderada (*cluster* B); insuficiente, oscilando atividade física fraca (*cluster* C); adequada, com aumento da atividade física moderada (*cluster* D).

Na Tabela 2, é possível observar o perfil dos homens situados em cada *cluster* das trajetórias de intensidade de atividade física, segundo as variáveis sociodemográficas e comportamentais. Nota-se que os homens situados no *cluster* C, que representam 6,2% do total, apresentaram maior média de renda, são mais jovens (72,1% entre 35 e 54 anos), tem escolaridade mais alta, nunca fumaram e apresentam um estado nutricional normal. Além disso, mais da metade dos homens situados nesse *cluster* referiram consumir diariamente verduras e frutas.

**Tabela 2:** Perfil dos homens situados nos *clusters* de trajetórias de intensidade de atividade física, segundo variáveis sociodemográficas e comportamentais. *Estudo Longitudinal de Saúde do Adulto* (ELSA-Brasil), 2008-2019 (N = 4.892).

Variável	*Clusters* * (%)				
	A [n = 3.738; 76,4%]	B [n = 611; 12,5%]	C [n = 305; 6,2%]	D [n = 120; 2,5%]	E [n = 11; 2,4%]
Renda *per capita* ** (média ± DP)	1.648,7 ± 1.311,4	1.691,0 ± 1.301,4	1.972,1 ± 1.526,2	1.814,0 ± 1.527,0	1.686,8 ± 1.359,7
Faixa etária (anos) **					
35-44	26,3	14,9	31,8	7,5	24,6
45-54	41,1	37,2	39,3	35,1	44,9
55-64	24,3	34,5	20,1	39,1	23,7
≥ 65	8,3	13,4	8,8	18,3	6,8
Ocupação **					
Aposentado	12,4	20,1	13,4	34,9	10,2
Ativo	87,6	79,9	86,6	65,1	89,8
Escolaridade **					
Baixa escolaridade	46,5	51,2	32,1	55,1	52,4
Alta escolaridade	53,5	48,8	67,9	44,9	47,6
Situação conjugal					
Casado	82,9	82,6	83,3	75,8	85,6
Não casado	17,1	17,4	16,7	24,2	14,4
Tabagismo **					
Ex-fumante	33,5	39,3	32,8	47,5	47,4
Fumante	13	11,1	9,3	13,3	11,1
Nunca fumou	53,5	49,6	57,9	39,2	41,5
Consumo de álcool					
No passado	19,5	18,5	15,3	25,1	16,1
Atual	75,9	77,9	79,4	73,3	78,8
Nunca consumiu	4,6	3,6	5,3	1,7	5,1
Estado nutricional **					
Normal	34,5	31,3	42,3	30,8	31,3
Sobrepeso	44,2	47,5	44,6	57,4	50,1
Obeso	21,3	21,2	13,1	11,8	18,6
Consumo diário de verduras					
Sim	45,4	47,9	52,6	47,5	42,4
Não	54,6	52,1	47,4	52,5	57,6
Consumo diário de frutas **					
Sim	45,9	54,9	54,7	50	44,9
Não	54,1	45,1	45,3	50	55,1

DP: desvio padrão.

* Insuficiente constante (*cluster* A); insuficiente, com aumento da atividade física fraca (*cluster* B); adequado, com aumento da atividade física forte (*cluster* C); insuficiente, com redução de atividade física fraca e aumento da moderada (*cluster* D); adequado, oscilando atividade física moderada e forte (*cluster* E).

** p < 0,05 (teste qui-quadrado para variáveis categóricas o teste t de Student para a variável renda).

Já as mulheres do *cluster* D, que representam 8,6% do total, apresentaram maior renda (2.207 ± 1.624) e escolaridade, e maior proporção que nunca haviam fumado e apresentavam um estado nutricional normal. Diferente dos homens, as mulheres desse cluster eram mais velhas (84,9% acima de 45 e 64 anos), em maior proporção aposentadas e apresentavam menor consumo diário de frutas (Tabela 3).

**Tabela 3:** Perfil das mulheres situadas nos *clusters* de trajetórias de intensidade de atividade física, segundo variáveis sociodemográficas e comportamentais. *Estudo Longitudinal de Saúde do Adulto* (ELSA-Brasil), 2008-2019 (N = 6.370).

Variáveis	*Clusters* * (%)			
	A [n = 3.806; 59,7%]	B [n = 1.202; 18,9%]	C [n = 815; 12,8%]	D [n = 547; 8,6%]
Renda *per capita* ** (média ± DP)	1.676,0 ± 1.411,0	1.930,6 ± 1.507,5	1.965,8 ± 1.605,4	2.207,0 ± 1.624,0
Faixa etária (anos) **				
35-44	25,3	23,6	15,7	15,1
45-54	41,9	40,1	43,3	37,3
55-64	25,9	28,8	31,4	34,1
≥ 65	6,9	7,5	9,6	13,5
Ocupação **				
Aposentado	18,7	22,1	23,3	27,6
Ativo	81,3	77,9	76,7	72,4
Escolaridade **				
Baixa escolaridade	47,7	39,7	43,3	30,5
Alta escolaridade	52,3	60,3	56,7	69,5
Situação conjugal				
Casado	55,9	51,1	53,6	53,1
Não casado	44,1	48,9	46,4	46,9
Tabagismo **				
Ex-fumante	24,5	26,6	26,7	24,5
Fumante	12,5	9,8	10,1	10,4
Nunca fumou	63	63,6	63,2	65,1
Consumo de álcool				
No passado	20,5	18,2	18,8	16,5
Atual	64,3	67,7	66,4	70,6
Nunca consumiu	15,2	14,1	14,8	12,9
Estado nutricional **				
Normal	38,1	40,8	41,7	44,1
Sobrepeso	35,8	36,9	35,1	34,6
Obeso	26,1	22,2	23,2	21,3
Consumo diário de verduras				
Sim	53,5	62,5	63,9	62,7
Não	46,5	37,5	36,1	37,3
Consumo diário de frutas **				
Sim	60,3	70,5	71,5	70,4
Não	39,7	29,5	28,5	29,6

DP: desvio padrão.

* Insuficiente constante (*cluster* A); insuficiente, com aumento da atividade física moderada (*cluster* B); insuficiente, oscilando atividade física fraca (*cluster* C); adequada, com aumento da atividade física moderada (*cluster* D).

** p < 0,05 (teste qui-quadrado para variáveis categóricas o teste t de Student para a variável renda).

**Figura 4:**
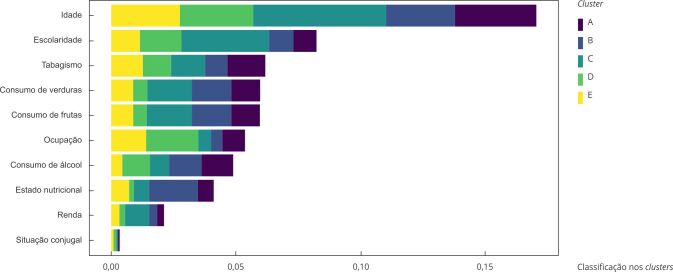
Classificação de importância dos preditores acordo com a média dos valores absolutos do SHAP para os homens. *Estudo Longitudinal de Saúde do Adulto* (ELSA-Brasil), 2008-2019.

A Figura 4 mostra a classificação de importância dos preditores de acordo com a média dos valores absolutos do SHAP para os homens. Observa-se que idade e escolaridade foram os preditores de maior importância para a classificação nos *clusters*, enquanto renda e situação conjugal foram as variáveis de menor importância. Para o *cluster* C, idade e a escolaridade também foram os preditores de maior importância, seguidos por consumo diário de frutas e verduras.

SHAP: *SHapley Additive exPlanations*. Nota: insuficiente constante (*cluster* A); insuficiente, com aumento da atividade física fraca (*cluster* B); adequado, com aumento da atividade física forte (*cluster* C); insuficiente, com redução de atividade física fraca e aumento da moderada (*cluster* D); adequado, oscilando atividade física moderada e forte (*cluster* E).

**Figura 5:**
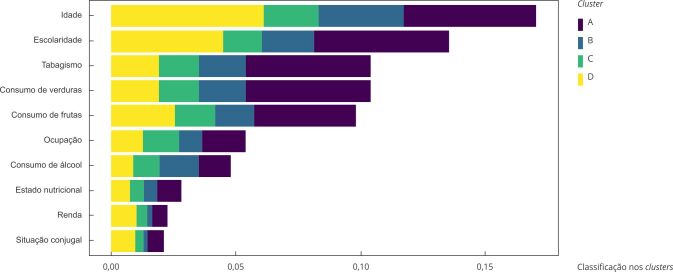
Classificação de importância dos preditores acordo com a média dos valores absolutos do SHAP para as mulheres. *Estudo Longitudinal de Saúde do Adulto* (ELSA-Brasil), 2008-2019.

Entre as mulheres, idade e escolaridade também foram os preditores de maior importância para a classificação nos *clusters*, enquanto renda e ocupação foram os de menor importância. Para o cluster D, idade, escolaridade e estado nutricional foram os preditores de maior importância (Figura 5).

SHAP: *SHapley Additive exPlanations*. Nota: insuficiente constante (cluster A); insuficiente, com aumento da atividade física moderada (cluster B); insuficiente, oscilando atividade física fraca (cluster C); adequada, com aumento da atividade física moderada (cluster D).

**Figura 6:**
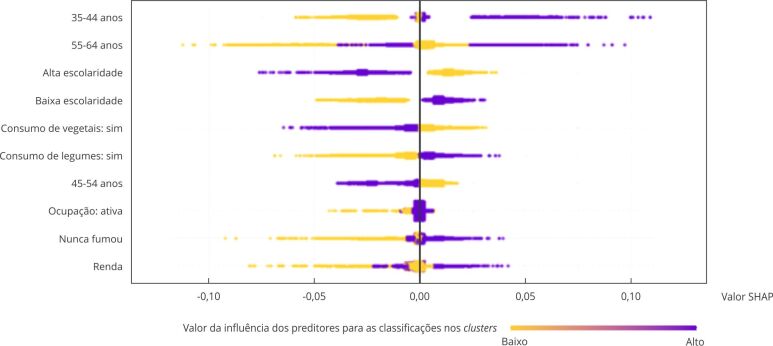
Influência dos preditores para a classificação no cluster de trajetória adequada, com aumento de atividade física forte (*cluster* C) em homens. Estudo Longitudinal de Saúde do Adulto (ELSA-Brasil), 2008-2019.

Nas Figuras 6 e 7 é possível observar a influência dos preditores para a classificação no *cluster* C em homens e no *cluster* D em mulheres, respectivamente. Para a direita do eixo y, é a influência positiva, e quanto mais escura a cor, maior a influência do preditor. Para os homens, observa-se que a faixa etária entre 35 e 44 anos, a escolaridade e o consumo diário de frutas e legumes foram os preditores de maior influência para pertencer ao *cluster* C, enquanto, para as mulheres, foi a fixa etária entre 55 e 64 anos, a escolaridade e ter estado nutricional normal.

SHAP: *SHapley Additive exPlanations*.

**Figura 7:**
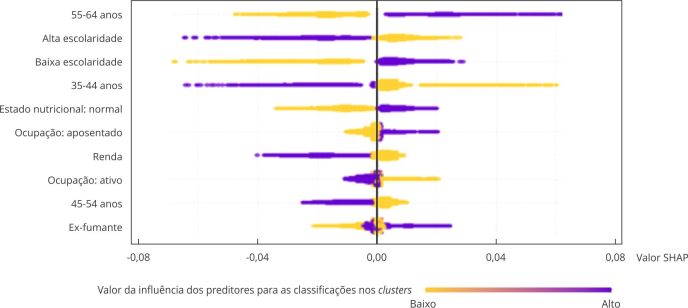
Influência dos preditores para a classificação no cluster de trajetória adequada, com aumento de atividade física moderada (*cluster* D) em mulheres. Estudo Longitudinal de Saúde do Adulto (ELSA-Brasil), 2008-2019.

SHAP: *SHapley Additive exPlanations*.

## Discussão

Este estudo identificou *clusters* de trajetórias de intensidade de atividade física em adultos brasileiros durante um período de seguimento de dez anos. Trajetórias distintas de atividade física foram observadas entre homens e mulheres, bem como diferenças no perfil sociodemográfico e comportamental dos participantes localizados em cada *cluster* e seus preditores. Em ambos os sexos, menos de 10% dos participantes apresentaram trajetória de atividade física adequada, sendo idade e escolaridade importantes preditores para a manutenção de atividade física recomendada. Participantes com atividade física adequada apresentavam maior renda e escolaridade, mais consumo diário de frutas e verduras e não eram tabagistas.

Para ambos os sexos, a maior parte dos participantes foi classificada nos *clusters* de trajetórias “insuficiente constante” (76,4% e 59,7% para homens e mulheres, respectivamente), dado que gera preocupação. Em relatório publicado em 2022, a OMS alertou que, entre 2020 e 2030, 500 milhões de pessoas desenvolverão doenças cardiovasculares, obesidade, diabetes, câncer, hipertensão, depressão e ansiedade decorrentes da inatividade física ^27^. Ilustrando a importância da prática da atividade física ao longo do tempo, Mok et al. ^4^ investigaram as associações entre trajetórias da atividade física e mortalidade por todas as causas, doenças cardiovasculares e câncer. Os pesquisadores avaliaram 14.599 homens e mulheres com idade entre 40 e 79 anos, e, após 12,5 anos de acompanhamento, encontraram associação inversa entre prática de atividade física e os desfechos analisados, concluindo que, independentemente da atividade física feita no passado, benefícios substanciais à saúde ocorrem com aumento da atividade física ao longo do tempo. Reforçando esses achados, Lee et al. ^28^ investigaram as associações entre trajetórias de atividade física no lazer e risco de mortalidade por todas as causas. Os pesquisadores analisaram dados de 21.211 participantes com idade entre 18 e 90 anos, que foram acompanhados por aproximadamente 17 anos, e concluíram que aqueles com trajetória média/estável ou crescente tiveram um risco significativamente menor de mortalidade por todas as causas. Portanto, o aumento da prática de atividade física ao longo do tempo é essencial para a saúde pública.

Neste estudo, os homens apresentaram maior tempo e intensidade de atividade física, resultado também relatado por Lounassalo et al. ^10^, que, em estudo de revisão sistemática incluindo 27 artigos sobre trajetórias de atividade física e fatores relacionados ao longo da vida, encontraram trajetórias da atividade física mais ativas entre os homens. Esses achados são parcialmente explicados pelas diferenças biológicas existentes entre homens e mulheres. Homens têm mais massa muscular, maior volume nas fibras musculares e maior capacidade de transporte e utilização de oxigênio; já as mulheres apresentam maior percentual de gordura corporal, menor força muscular e menor capacidade de produção de energia ^29^. A atividade física entre as mulheres também é influenciada negativamente pelas expectativas da sociedade e pelas normas culturais tais como tempo disponível para a prática de atividade física no lazer, imagem corporal, padrões sociais de beleza, deveres familiares e segurança ^30^.

Também verificamos que a proporção de mulheres no *cluster* de trajetória adequada era menor do que a dos homens para a faixa etária mais jovem (35 a 44 anos). Isso pode ser explicado pelo fato de a mulher estar numa fase da vida caracterizada por muitas funções, tais como a maternidade ^31^. Cabe mencionar que neste estudo foi considerada apenas a atividade física no tempo livre. Em estudo conduzido por Hallal et al. ^32^, não foram encontradas diferenças entre homens e mulheres quando a atividade física doméstica também foi considerada. Portanto, a atividade física doméstica, realizada predominante por mulheres, é importante em estudos sobre atividade física que considerem as diferenças entre os sexos.

Os homens e mulheres situados nos *clusters* de melhor adequação de atividade física apresentaram maior escolaridade e maior renda, corroborando os resultados encontrados em estudo brasileiro ^33^. Ao serem analisados a prevalência e os fatores associados à prática de atividade física no lazer em adultos, a conclusão foi que a escolaridade foi associada à maior chance de atividade física no lazer. A maior escolaridade favorece o acesso às ocupações com maior renda, maior literacia em saúde e acesso às melhores condições de vida e aos serviços de saúde, favorecendo também ambientes mais adequados para a prática de atividade física no lazer ^34^.

Para ambos os sexos, idade e escolaridade foram os preditores de maior importância para a classificação nos *clusters*. Cabe ressaltar que estudos sobre fatores associados às trajetórias de atividade física que se encontram disponíveis na literatura ^10^ ^,^ ^11^ ^,^ ^33^ não utilizaram a técnica presente neste estudo, sendo este um aspecto inovador do tema.

Este estudo se destaca por pontos fortes. Foram utilizados dados de uma coorte de adultos residentes em regiões distintas do Brasil, com alto rigor na garantia e controle de qualidade na coleta de dados. Além disso, o estudo considerou as informações sobre atividade física em três momentos do tempo, diferenciando-se da maioria dos estudos longitudinais prévios, que analisaram a atividade física em dois pontos do tempo. O estudo também analisou, de forma inovadora, as diferentes intensidades da atividade física ao longo do tempo e permitiu identificar a influência das variáveis sociodemográficas e comportamentais em cada *cluster* de trajetória encontrado, possibilitando reconhecer os fatores preditores dos *clusters*.

No entanto, algumas limitações podem ter influenciado os achados. A utilização de questionários para aferir a atividade física pode levar ao viés de informação. Todavia, a literatura aponta que o IPAQ tem alta confiabilidade, e o uso do domínio do tempo livre da versão longa (utilizada no presente estudo) é recomendado em estudos que visem documentar a atividade física na América Latina ^35^. Ademais, estudos recentes que investigaram níveis de atividade física utilizaram o IPAQ, reforçando sua validade ^36^ ^,^ ^37^. Outra limitação do estudo é o fato de as variáveis comportamentais terem sido coletadas apenas na linha de base, não sendo possível analisar a associação de suas mudanças ao longo do tempo com a manutenção de intensidade de atividade física. Por fim, este estudo não considerou doenças ou motivos de saúde que estão associados à manutenção de atividade física ao longo do tempo, tais como doenças cardiovasculares, hipertensão, depressão, artrite, bronquite ^10^ ^,^ ^38^. Essas doenças influenciam a prática de atividade física ao longo do tempo e podem explicar parcialmente as mudanças que ocorrem na prática de atividade física. Porém, esta análise enfatizou questões que devem ser consideradas na implementação de políticas públicas destinadas a promover a prática de atividade física ao longo do tempo.

## Conclusão

Este é o primeiro estudo longitudinal brasileiro que identificou *clusters* de trajetórias de atividade física, explorando as diferentes intensidades em três momentos do tempo, além de ter identificado seus preditores. Os resultados deste estudo visam reforçar a necessidade de implementação de programas e políticas públicas destinados à prática e manutenção da atividade física recomendada para adultos, integrados a outras estratégias de promoção de saúde, tais como ampla divulgação para toda a população dos benefícios da atividade física, criação de espaços destinados à sua prática e adaptados às diferentes faixas etárias e condições de vida, incentivo ao consumo de frutas e verduras e cessação do tabagismo. Além disso, são necessárias campanhas que superem as barreiras existentes entre as mulheres, para que elas tenham maior disponibilidade de tempo para a prática de atividade física no tempo livre.
