# Supplementary material for: Cluster predictors of trajectories of leisure-time physical activity intensity in men and women from ELSA-Brasil
Source: Cad Saude Publica. 2025 May 19;41(4):e00132924. [Article in Portuguese] doi: 10.1590/0102-311XPT132924 (PMC12091857; doi:10.1590/0102-311XPT132924)
Supplement: Supplementary file 1 [file 1678-4464-csp-41-04-PT132924-s.pdf]

Material Suplementar

Tabela S1 Medidas de desempenho de floresta aleatória multiclasse de acordo com o sexo e *clusters* das trajetórias de intensidade de atividade física. *Estudo Longitudinal de Saúde do Adulto* (ELSA-Brasil), 2008-2019.

**Medidas**	**Homens**	**Mulheres**
Acurácia (média±DP)	0.45±0.07	0.51±0.02
Precisão (média±DP)	0.63±0.02	0.43±0.01
Revocação (média±DP)	0.45± 0.07	0.51±0.02
F1 - Pontuação (média±DP)	0.52±0.06	0.46±0.01
